# Multimodal Deep Learning and Knowledge‐Enhanced Intelligent Decision Support System for Pipeline Embolization Device Size Selection in Intracranial Aneurysm Treatment

**DOI:** 10.1002/cns.71047

**Published:** 2026-07-22

**Authors:** Zhihong Wen, Shengli Guo, Yulin Peng, Yakun Chen, Luokai Huangfu, Hao Zhao, Hao Gao, Taoyi Ni, Jianning Zhang, Xiangpeng Liu, Jiayu Liu, Yongping Liang

**Affiliations:** ^1^ College of Information, Mechanical & Electrical Engineering Shanghai Normal University Shanghai China; ^2^ Department of Neurosurgery, the First Medical Center Chinese PLA General Hospital Beijing China; ^3^ Department of Neurosurgery, the Six Medical Center Chinese PLA General Hospital Beijing China

**Keywords:** intelligent decision support system, intracranial aneurysm, knowledge enhancement, multimodal deep learning, pipeline embolization device

## Abstract

**Objective:**

This study aimed to develop an end‐to‐end intelligent decision support system, NeurAneuNet, to automate the selection of Pipeline Embolization Device (PED) size and landing zones for intracranial aneurysm treatment, thereby reducing reliance on operator experience and improving planning consistency.

**Methods:**

NeurAneuNet integrates multimodal deep learning with knowledge enhancement. The system processes three‐dimensional rotational angiography (3DRA) images using a dual‐path attention U‐Net++ for aneurysm segmentation, extracts knowledge‐augmented geometric and clinical features, fuses five feature modalities via tensor decomposition, and predicts optimal PED sizing and landing zones with a high‐order Kolmogorov–Arnold Network (KAN). A dataset of 600 aneurysms (including 210 PED‐treated cases) was used for model development and validation, with an independent clinical cohort of 21 cases employed to assess real‐world utility.

**Results:**

NeurAneuNet achieved a Dice coefficient of 0.874 ± 0.03 for aneurysm segmentation, a PED size classification accuracy of 91.8%, and a diameter prediction error of 0.24 ± 0.10 mm. In the independent test, its primary recommendation agreed with the expert consensus in 95.2% of cases. When assisting clinicians, the system reduced planning time by 44.8% and significantly lowered subjective cognitive workload, as assessed by the National Aeronautics and Space Administration Task Load Index (NASA‐TLX) score (from 33 ± 8 to 21 ± 5).

**Conclusion:**

NeurAneuNet demonstrates clinically relevant accuracy and efficiency in automating PED treatment planning, providing robust, intelligent support for intracranial aneurysm interventions. The system provides a promising foundation for developing a generalizable AI‐driven decision support framework in complex medical scenarios.

## Graphical abstract

This study presents NeurAneuNet, a multimodal deep learning framework designed to automate the selection of PED sizes for intracranial aneurysm treatment. The proposed end‐to‐end system integrates image segmentation, feature fusion, and knowledge‐based enhancement, delivering clinically accurate and efficient performance that effectively supports physician decision‐making.

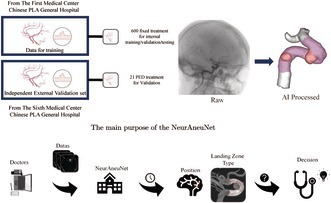

## 1 Introduction

Intracranial aneurysms are common and potentially fatal cerebrovascular conditions, as their rupture leads to subarachnoid hemorrhage (SAH), a critical neurosurgical emergency with high rates of mortality and morbidity. Epidemiological studies report that aneurysmal SAH has a 30‐day mortality rate of 40%–50%, and about one‐third of survivors sustain permanent neurological deficits [1]. Although advances in imaging have markedly improved aneurysm detection, precise morphological characterization and the formulation of optimal, individualized treatment plans remain major clinical challenges.

Pipeline Embolization Device (PED), as the first flow diverter, has transformed the treatment paradigm for complex intracranial aneurysms [2, 3]. The efficacy of PED therapy hinges on precise size selection and accurate placement within the parent vessel, factors that critically influence clinical outcomes and complication risks. Current clinical workflows remain heavily reliant on operator expertise, presenting three major challenges: (1) traditional segmentation and measurement of aneurysms and parent vessels rely on manual or semi‐automated methods, which are time‐consuming and exhibit significant interobserver variability [4]; (2) manual measurement of key anatomical parameters lacks consistency and reproducibility, undermining the stability of clinical decisions; and (3) optimal PED sizing and landing‐zone determination require the complex integration of multidimensional parameters—including aneurysm morphology, parent vessel geometry, and hemodynamic characteristics—a synthesis that is difficult to perform efficiently, even for experienced clinicians.

**FIGURE 1:**
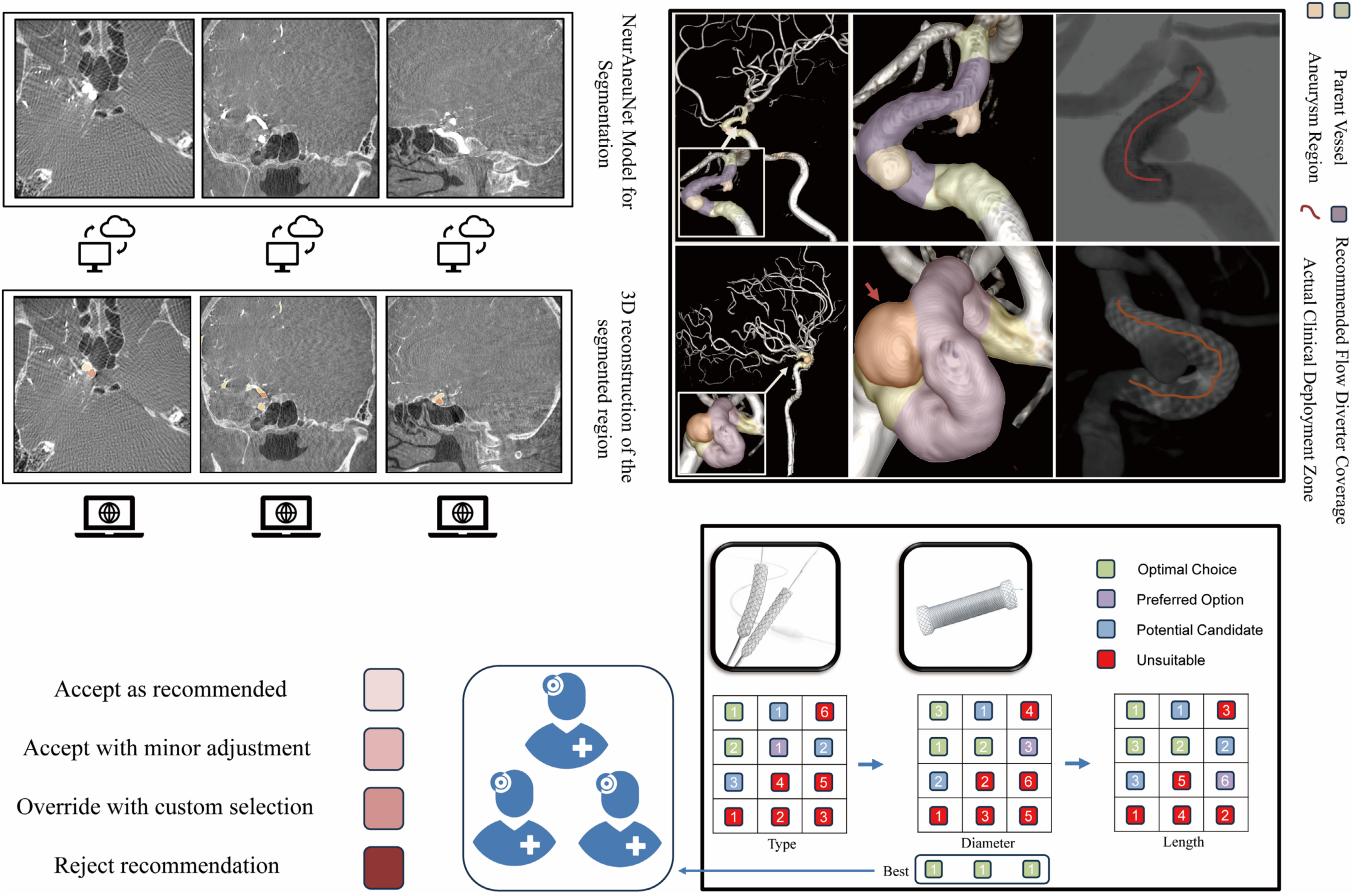
Schematic of NeurAneuNet's end‐to‐end intelligent decision workflow: The figure illustrates dual‐path attention U‐Net++ aneurysm segmentation, PED landing‐zone coverage, and three‐dimensional reconstruction of landing zones, alongside PED size, diameter, and length recommendations derived from multimodal fusion and KAN, provided for clinician reference.

Breakthroughs in deep learning for medical image analysis offer promising avenues to address these challenges [5, 6]. Notably, Han et al. recently proposed a multiscale attention network via topology learning for cerebral vessel segmentation in angiography images, demonstrating that multiscale attention mechanisms and topology‐aware constraints can improve the continuity and completeness of vascular structure extraction [7]. This study provides valuable methodological support for attention‐guided and topology‐preserving segmentation in neurovascular image analysis. Inspired by these methodological advances, we further aimed to extend neurovascular image analysis beyond vascular segmentation toward a comprehensive decision‐support framework that integrates segmentation, knowledge‐enhanced feature extraction, multimodal fusion, and treatment planning. Given that imaging features alone may be insufficient to capture the complexity of clinical decision‐making, incorporating multimodal data and domain‐specific knowledge is essential. To bridge these gaps, we developed NeurAneuNet—an end‐to‐end intelligent decision support system comprising five core modules: data preprocessing, segmentation, knowledge‐enhanced feature extraction, multimodal fusion based on tensor decomposition, and decision‐making utilizing a high‐order Kolmogorov–Arnold Network (KAN). Specifically, NeurAneuNet employs a dual‐path attention U‐Net++ for segmentation, knowledge‐augmented geometric feature extraction, tensor decomposition for multimodal fusion, and KAN for high‐order nonlinear mapping. This design automates the complete workflow from three‐dimensional rotational angiography (3DRA) analysis to PED sizing and landing‐zone determination (Figure 1), aiming to enhance treatment safety and efficacy while reducing reliance on operator experience.

## 2 Methods

### 2.1 Datasets

This study enrolled patients with intracranial aneurysms located between the cavernous and communicating segments of the internal carotid artery, who were treated in the Department of Neurosurgery, First Medical Center of Chinese People's Liberation Army (PLA) General Hospital between September 2017 and February 2025. Of these, 210 aneurysms were treated with PEDs (Medtronic's Pipeline Flex or Pipeline Shield). The dataset was divided into two subsets: (1) 390 aneurysms not treated with PEDs, used to develop segmentation and parent vessel measurement models; and (2) 210 PED‐treated aneurysms, used to validate the measurement model and to develop, validate, and test models for PED sizing and landing‐zone determination. The PED‐treated cases were further split into training (*n* = 147), validation (*n* = 21), and testing (*n* = 42) sets at a ratio of 7:1:2.

For each case, the following data were collected: pre‐ and post‐operative 3DRA images (512 × 512 matrix, slice thickness 0.625–1.0 mm, voxel resolution 0.5 × 0.5 × 0.8 mm), standardized with a window width of 800 Hounsfield Units (HU) and level of 200 HU. Clinical phenotypic data included aneurysm location, neck width, parent vessel length, and the clinically determined optimal proximal and distal landing zones for PED placement. Treatment data comprised the PED size selected by the surgeon, along with the actual proximal and distal landing zones achieved during the procedure.

### 2.2 Model Development

#### 2.2.1 Pre‐Processing

Image preprocessing: Preoperative 3DRA images, acquired with a 512 × 512 matrix and a slice thickness of 0.625–1.0 mm, possessed an approximate voxel resolution of 0.5 × 0.5 × 0.8 mm. For each voxel *x*, local adaptive normalization was applied [8]:

$$I\mathrm{norm}\left(x\right)=\frac{I\left(x\right)-\mathrm{\mu N}\left(x\right)}{\sqrt{\sigma_{N\left(x\right)}^{2}+\varepsilon }}$$

where $\mathrm{\mu N}\left(x\right)$, $\mathrm{\sigma N}\left(x\right)$ denote the mean and standard deviation, respectively, within a local window centered at voxel *x*. The term ε = 10^−6^ is a small constant incorporated to prevent numerical instability.

Multiscale enhancement: Normalized images were processed using a three‐level Gaussian pyramid, with up sampling and down sampling operations performed via cubic B‐spline interpolation. Anisotropic diffusion filtering was subsequently applied to suppress noise while preserving critical edge information.

Online data augmentation: The training dataset was augmented online through a series of transformations including random rotations (±20°), scaling (factor range: 0.9–1.1), elastic deformations, and intensity perturbations. Furthermore, targeted oversampling was implemented for rare cases involving minute or giant aneurysms to enhance model generalizability.

#### 2.2.2 Segmentation Network

Dual‐path attention‐residual U‐Net++: The segmentation network employed a dual‐path architecture [9, 10, 11], which comprised a main path for deep feature extraction and a parallel auxiliary path for lightweight perturbation analysis. The outputs from these respective paths were subsequently fused using a learnable weight, *w*, as follows:

$$S=w\cdot S\text{main}+\left(1-w\right)\cdot S\mathrm{aux},w\approx 0.8\left(\text{init}\right)$$

Dynamic convolution: For the input feature $X\in R^{C\times H\times W}$ groups of convolution kernels $\left\{\kappa_{k}\right\}$ and attention weights $\left\{\alpha_{k}\right\}$ are employed:

$$Y=\sum k=1^{K}\alpha_{k}\left(\kappa_{k}*X\right),\alpha =\text{soft}\max\left(\mathrm{MLP}\left(\mathrm{GAP}\left(X\right)\right)\right)$$

Channel and spatial attention: The Convolutional Block Attention Module (CBAM) was incorporated at each stage of the decoder. Channel attention maps were generated via global average pooling and a multi‐layer perceptron (MLP), while spatial attention maps were produced using subsequent convolutional layers. This dual attention mechanism was employed to selectively amplify feature responses in foreground regions.

Adversarial training: To further refine segmentation performance, the segmentation network (Generator, G) was trained adversarially against a discriminator network (D) using the Wasserstein Generative Adversarial Network (WGAN) with Gradient Penalty framework [12]. A gradient penalty coefficient $\lambda_{\mathrm{gp}}$ = 10 was applied. This adversarial scheme was designed to enhance the fidelity of the segmentation maps and improve the robustness of identifying and delineating small‐volume aneurysms.

#### 2.2.3 Multimodal Fusion and Decision Network

Image features $Z_{\mathrm{img}}\in R^{d_{1}}$: Bottleneck features were extracted from the primary segmentation network, followed by spatial/channel attention mechanisms and global pooling [13, 14, 15, 16].

Geometric and knowledge‐enhanced features $Z_{\mathrm{img}}\in R^{d_{1}}$: Local diameter, curvature, and other relevant parameters were computed from centerline point sets using the Marching Cubes algorithm. Knowledge graph embeddings (e), obtained via ComplEx tensor decomposition, were subsequently fused using a bidirectional Gated Recurrent Unit (GRU) network incorporating an attention mechanism.

Tabular and temporal features $Z_{\mathrm{tab}}\in R^{d_{3}},Z_{\mathrm{tmp}}\in R^{d_{4}}$: Clinical variables were mapped using fully connected networks, while temporal data pertaining to vessel diameter were encoded using one‐dimensional (1D) convolutional layers followed by pooling.

Multimodal fusion: The five distinct feature modalities were combined via outer product to construct a high‐order $\chi =Z_{\mathrm{img}}⊗Z_{\mathrm{geo}}⊗Z_{\text{know}}⊗Z_{\mathrm{tab}}⊗Z_{\mathrm{tmp}}$, which was then subjected to Canonical Polyadic (CP) decomposition [17, 18]:

$$\chi \approx \sum_{r=1}^{R}a_{r}\circ b_{r}\circ c_{r}\circ d_{r}\circ e_{r},R=50$$

Following the extraction of interaction vectors for each modality pair, these vectors were weighted, aggregated, and processed through layer normalization to yield the fused features $\left(z_{\mathrm{fus}}\right)$.

Based on the Kolmogorov‐Arnold representation theorem [19, 20, 21], polynomial basis functions ({$\phi_{q}$}) of orders one to three were constructed for the input $\left(z_{\mathrm{fus}}\in R^{n}\right)$. After fusion, significant interaction terms were selected via sparse attention and aggregated:

$$f\left(z_{\mathrm{fus}}\right)\approx \sum_{q=1}^{Q}\beta_{q}\phi_{q}{\left(w_{q}z_{\mathrm{fus}}+b_{q}\right)}_{r},Q=2n+1$$

The resultant features were then mapped to three distinct task‐specific output branches:

Device model classification: Cross‐entropy loss ($L_{\mathrm{cls}}$).

Diameter/length regression: Negative Log‐Likelihood (NLL) loss from a Mixture Density Network ($L_{\mathrm{reg}}$).

Landing zone prediction: $L_{\text{total}}$ Geometric constraint loss ($L_{\mathrm{pos}}$). The overall loss function was formulated as an adaptive balance of these individual losses, weighted by learnable parameters ({$\lambda_{i}$}):

$$L=\lambda_{1}L_{\mathrm{cls}}+\lambda_{2}L_{\mathrm{reg}}+\lambda_{3}L_{\mathrm{pos}}$$

All network modules were trained jointly in an end‐to‐end fashion.

### 2.3 Experimental Setup

All experiments were conducted on a high‐performance computing cluster equipped with eight NVIDIA A100 Graphics Processing Units (GPUs) (80GB each), implemented using the PyTorch 1.12.0 framework. Model training employed a meticulously designed five‐stage strategy:

The Segmentation Pre‐training Stage (200 epochs) involved training the dual‐path U‐Net++ segmentation network. The optimization target was a composite loss function comprising Tversky loss, boundary loss, and Structural Similarity Index (SSIM) loss. This stage utilized the Adam with Weight Decay (AdamW) optimizer, a learning rate of 1 × 10^−4^, weight decay of 1 × 10^−5^, and a batch size of 4.

Next, in the Adversarial Training Stage (100 epochs), the main segmentation network was frozen, and the WGAN was trained. This stage used a learning rate of 5 × 10^−5^ and a batch size of 4.

The third stage was Geometric Feature Extraction and Knowledge Enhancement Training (150 epochs). Here, the geometric feature extraction and knowledge enhancement networks were trained using the segmentation results, with a learning rate of 8 × 10^−5^, weight decay of 2 × 10^−5^, and a batch size of 8.

Subsequently, the Multimodal Fusion and KAN Training stage (120 epochs) was performed. During this phase, components from the preceding stages were frozen, and the tensor decomposition fusion mechanism and the KAN were trained. The Rectified Adam (Radam) optimizer was employed with a learning rate of 5 × 10^−5^ and a batch size of 6.

Finally, End‐to‐End Fine‐tuning (50 epochs) was conducted. All modules were unfrozen for joint optimization, carried out with a learning rate of 2 × 10^−5^ and a batch size of 4.

The learning rate was scheduled using a cosine annealing strategy with $\eta_{\min}$ = 1 × 10^−7^. Model hyperparameters were tuned automatically via Bayesian optimization over 125 tested parameter combinations. The finally determined key parameters included: KAN basis function rank *r* = 50, *h* = 8 attention heads, feature embedding dimension *d* = 256, *C* = 3 Gaussian mixture components, and an adversarial weight $\lambda_{\mathrm{adv}}$ = 0.1. The complete model training required 18.3 days, with most of this time dedicated to the segmentation pre‐training and adversarial training stages. An early stopping strategy was implemented based on validation set performance, halting training if no improvement was observed for 10 consecutive epochs.

### 2.4 Clinical Application

To evaluate the clinical utility of the NeurAneuNet system and its impact on physician decision‐making, we recruited an independent test cohort of 21 PED‐treated aneurysm cases from the Sixth Medical Center of the Chinese PLA General Hospital. Detailed demographics, aneurysm location, and the clinically determined optimal PED size were collected for each case (Table 1). The study enrolled six neurointerventionalists, who were stratified into three cohorts based on their clinical seniority: senior (*n* = 2), intermediate (*n* = 2), and junior (*n* = 2). Each physician was required to select PED sizing for all 21 cases under two sequentially administered, blinded conditions: (1) No‐AI Condition: Physicians performed planning using conventional methods, relying solely on the provided 3D angiographic images and clinical data. (2) AI‐Assisted Condition: Physicians performed planning with the assistance of the NeurAneuNet system. The system provided automated segmentation, key morphometric measurements, and a list of its top three PED recommendations with corresponding confidence scores.

**TABLE 1:** Twenty‐one external test cases and the PED sizes recommended by the NeurAneuNet system.

Case	Age	Sex	Location	Optimal PED (mm × mm)	AI‐recommended PED (mm × mm)	Confidence level (%)
1	42	F	Ophthalmic Seg (R)	4.0 × 18	4.25 × 18	81.5
2	43	F	Paraclinoid Seg (L)	4.0 × 12	4.0 × 12	95.8
3	48	M	Ophthalmic Seg (L)	5.0 × 20	5.0 × 20	96.8
4	49	M	Ophthalmic Seg (R)	5.0 × 20	5.0 × 20	91.2
5	40	M	Paraclinoid Seg (R)	4.75 × 35	4.75 × 35	88.2
6	60	M	Ophthalmic Seg (R)	4.5 × 20	4.5 × 20	94.2
7	60	M	Ophthalmic Seg (L)	5.0 × 20	5.0 × 20	90.8
8	65	F	Ophthalmic Seg (L)	4.0 × 14	4.0 × 14	91.3
9	71	F	Communicating seg (R)	4.5 × 14	4.5 × 14	91.5
10	53	F	Paraclinoid Seg (R)	4.0 × 18	4.0 × 18	96.5
11	55	F	Ophthalmic Seg (L)	4.5 × 18	4.5 × 18	94.8
12	53	F	Ophthalmic Seg (L)	4.5 × 14	4.5 × 14	92.8
13	53	F	Ophthalmic Seg (R)	4.5 × 14	4.5 × 14	93.2
14	64	F	Communicating seg (R)	4.0 × 16	4.0 × 16	97.2
15	62	M	Ophthalmic Seg (R)	5.0 × 18	5.0 × 18	94.5
16	63	F	Cavernous seg (R)	5.0 × 35	5.0 × 35	88.3
17	62	M	Ophthalmic Seg (R)	4.0 × 20	4.0 × 20	82.4
18	65	F	Ophthalmic Seg (R)	4.75 × 16	4.75 × 16	92.5
19	62	F	Cavernous seg (R)	5.0 × 30	5.0 × 30	90.8
20	56	F	Ophthalmic Seg (R)	4.0 × 20	4.0 × 20	93.6
21	56	M	Ophthalmic Seg (L)	5.0 × 16	5.0 × 16	89.3

Abbreviation: PED, pipeline embolization device.

The optimal PED for each case (serving as the reference standard) was determined via consensus discussion among three senior neurointerventionists, based on a comprehensive postoperative assessment of device wall apposition, length, and diameter. NeurAneuNet's primary recommendation was then compared with this expert‐defined reference standard for quantitative evaluation. All accuracy‐related performance metrics, including PED recommendation accuracy and physician‐system agreement, were calculated using the top‐1 NeurAneuNet recommendation. The top three recommendations with confidence scores were provided for clinical interpretability and uncertainty management and were not used to inflate the reported performance.

The primary outcome was the planning time (in seconds) required to finalize a PED selection. Secondary outcomes included the subjective cognitive workload, measured using the National Aeronautics and Space Administration Task Load Index (NASA‐TLX), and the agreement between the physician's final PED selection and the optimal PED (Table 2). To account for potential learning effects, the order of the two conditions was randomized, and a sufficient washout period (2 weeks) was enforced between testing sessions.

**TABLE 2:** The impact of the NeurAneuNet system assistance on the planning efficiency, workload, and consistency of neurointerventional physicians.

Physician cohort	Planning time (s)		NASA‐TLX		Agreement	
	No‐AI	With‐AI	No‐AI	With‐AI	No‐AI	With‐AI
Senior (*n* = 2)	435 ± 112	361 ± 48	26 ± 4	16 ± 5	42/42	42/42
	(300–702)	(282–472)	(17–35)	(9–28)	(100%)	(100%)
Intermediate (*n* = 2)	647 ± 86	372 ± 51	32 ± 5	22 ± 4	34/42	40/42
	(505–832)	(311–492)	(24–46)	(18–35)	(80.9%)	(95.2%)
Junior (*n* = 2)	933 ± 76	381 ± 55	41 ± 6	24 ± 4	29/42	39/42
	(809–1131)	(312–512)	(28–52)	(15–36)	(69.0%)	(92.9%)
All physicians (*n* = 6)	672 ± 225	371 ± 51	33 ± 8	21 ± 5	105/126	121/126
	(300–1131)	(282–512)	(17–52)	(9–36)	(83.3%)	(96.0%)

Abbreviations: NASA ‐ TLX, National Aeronautics and Space Administration Task Load Index.

For clinical use, NeurAneuNet was designed as an assistive decision‐support system rather than an autonomous decision‐making tool. In addition to the primary recommendation, the system provides the top three PED recommendations with corresponding confidence scores. Predictions with high top‐1 confidence and a clear confidence margin from the alternative recommendations may be considered as supportive references for physician planning. Conversely, low‐confidence predictions, small confidence margins between the top‐ranked recommendations, or cases involving complex morphology, indistinct aneurysm boundaries, microaneurysms, marked vessel tortuosity, or lesions near device‐size transition thresholds should be flagged for mandatory expert review.

Based on the current validation results, we added a preliminary safety threshold: recommendations with a top‐1 confidence score below 85% should be regarded as uncertain and require mandatory expert review. This threshold is intended as a conservative safety trigger rather than a validated autonomous decision threshold and should be further calibrated in prospective multicenter studies.

## 3 Results

### 3.1 Overall Model Performance Analysis

**FIGURE 2:**
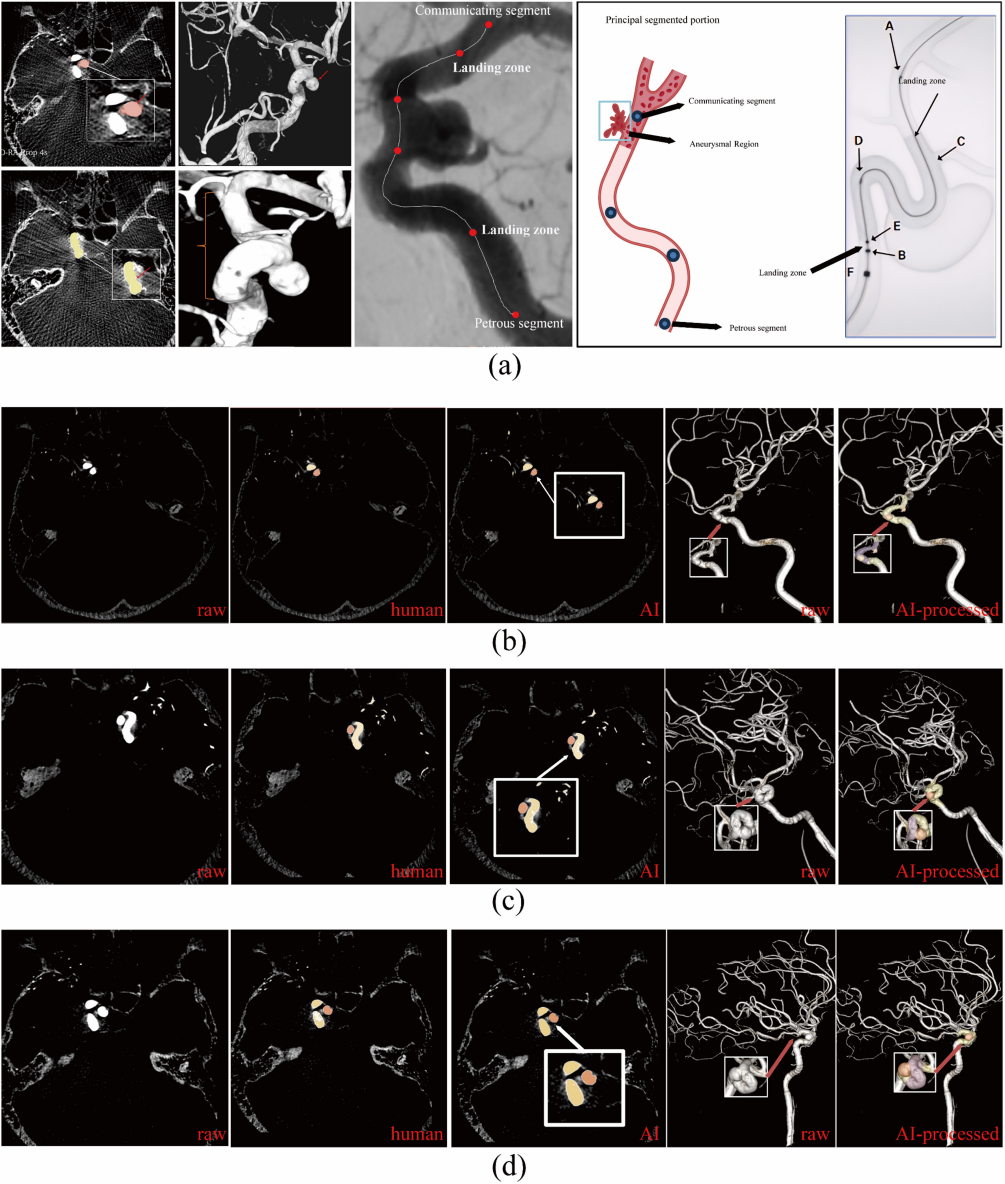
Qualitative visualization of NeurAneuNet aneurysm segmentation. (a) Schematic illustration of aneurysm and parent vessel segmentation. The left panel highlights the aneurysmal region, the middle panel shows the AI‐extracted aneurysm and key landing zones, and the right panel provides an anatomical diagram labeling the aneurysmal region, communicating segment, petrous segment, and landing zones. (b–d) Qualitative segmentation examples from three representative clinical cases. For each case, images are shown from left to right as the raw two‐dimensional slice, manual annotation, AI‐generated segmentation with a magnified region of interest, raw three‐dimensional volume‐rendered reconstruction, and AI‐processed three‐dimensional reconstruction.

NeurAneuNet achieved an overall Dice coefficient of 0.874 ± 0.03 (95% CI: [0.865, 0.883]) for aneurysm segmentation. Stratified by aneurysm size, the model recorded Dice coefficients of 0.748 ± 0.08 (95% CI: [0.723, 0.773]) for micro and small aneurysms (< 5 mm). Figure 2 presents qualitative segmentation examples from three representative clinical cases. For each case, raw images, manual annotations, and AI‐generated segmentations are shown on two‐dimensional slices and three‐dimensional volume‐rendered reconstructions, together with the corresponding raw and AI‐processed images for visual comparison.

**FIGURE 3:**
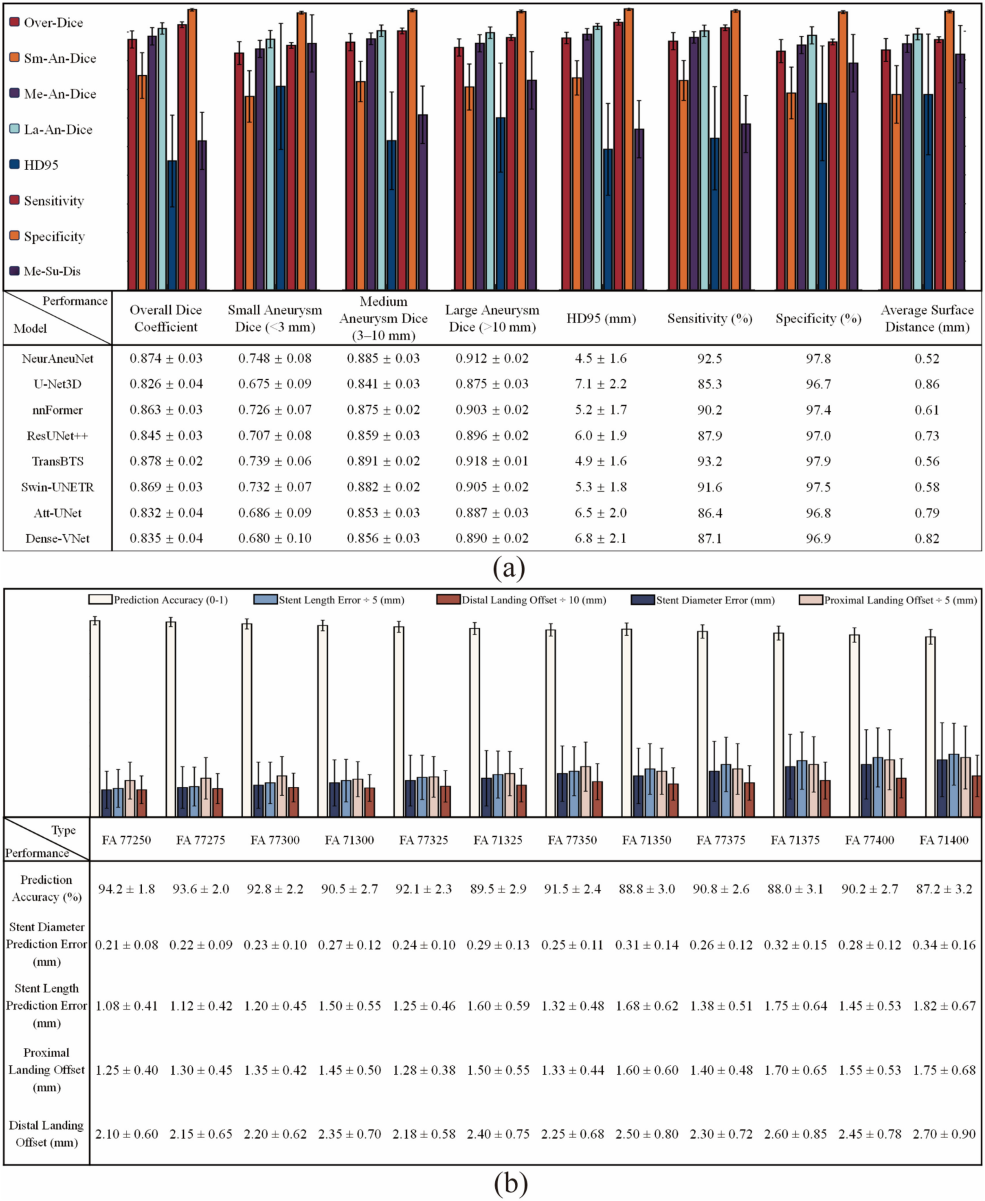
Performance summary of NeurAneuNet. (a) Aneurysm segmentation performance comparison: Overall Dice coefficient, Dice for small/medium/large aneurysms, HD95, sensitivity, specificity, and mean surface distance, with cross‐model comparisons highlighting NeurAneuNet's superiority. (b) PED size selection and parameter errors: Classification accuracy, diameter prediction error, length prediction error, proximal landing‐zone offset, and distal landing‐zone offset for 12 models.

**FIGURE 4:**
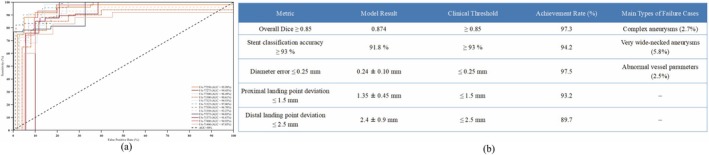
Performance evaluation and clinical threshold analysis of NeurAneuNet. (a) Receiver operating characteristic (ROC) curves for PED model classification across different device models, with the corresponding area under the curve (AUC) values shown in the legend. (b) Clinical threshold achievement analysis and main failure categories. This panel summarizes the predefined clinical thresholds, model results, threshold achievement rates, and corresponding major failure types for overall Dice coefficient, PED classification accuracy, diameter prediction error, proximal landing‐zone deviation, and distal landing‐zone deviation.

Boundary precision metrics included a 95th percentile Hausdorff Distance (HD95) of 4.5 ± 1.6 mm (95% CI: [4.02, 4.98]) and a mean surface distance of 0.52 ± 0.15 mm (95% CI: [0.474, 0.566]). Sensitivity was 92.5% (95% CI: [89.9%, 95.1%]), and specificity was 97.8% (95% CI: [96.6%, 99.0%]). Segmentation quality exceeded a Dice coefficient of 0.82 in 95% of test samples. For the clinically critical neck region, the model achieved a Dice coefficient of 0.865 ± 0.03 (95% CI: [0.856, 0.874]) for wide‐neck aneurysm neck boundary segmentation. PED size classification accuracy ranged from 87.2% to 94.2% (mean 91.8% (95% CI: [91.3%, 92.3%]); Figure 3a). Diameter prediction error was 0.24 ± 0.10 mm (95% CI: [0.209, 0.271]). Length prediction errors were 1.08–1.45 mm for short PEDs (10–20 mm) and 1.50–1.82 mm for long PEDs (25–35 mm; Figure 3b). Mean proximal landing‐zone offset was 1.52 ± 0.47 mm (95% CI: [1.378, 1.662]), and distal offset was 2.38 ± 0.69 mm (95% CI: [2.171, 2.589]). Stratified by PED diameter, proximal offsets were 1.18 ± 0.36 mm (95% CI: [1.071, 1.289]) for small‐diameter PEDs (2.50–3.00 mm), 1.42 ± 0.44 mm (95% CI: [1.287, 1.553]) for medium‐diameter PEDs (3.25–3.75 mm), and 1.78 ± 0.55 mm (95% CI: [1.614, 1.946]) for large‐diameter PEDs (4.00–5.00 mm). For PED size classification, the mean Area Under Curve (AUC) was 95.14%, with AUCs of 95.99%, 96.49%, and 97.06% for common models FA77250, FA77300, and FA71325, respectively, and a minimum AUC of 87.85% for FA71400 (Figure 4a).

To further characterize segmentation accuracy beyond voxel‐overlap metrics, we performed a systematic comparison of key morphological parameters between AI‐generated and manually delineated segmentations. Four clinically relevant parameters were evaluated: maximum diameter, neck width, neck‐to‐dome ratio, and aspect ratio. On the internal test set (*n* = 42), intraclass correlation coefficient (ICC) values ranged from 0.886 to 0.941, with a mean bias of +0.09 mm (95% limits of agreement (LoA): −0.68 to +0.86 mm) for maximum diameter and +0.13 mm (95% LoA: −0.72 to +0.98 mm) for neck width, both within clinically acceptable limits for PED size selection. On the independent external validation cohort (*n* = 21), ICC values ranged from 0.872 to 0.928, marginally lower than those of the internal test set, consistent with expected performance variation in external validation. The mean bias for maximum diameter was +0.12 mm (95% LoA: −0.82 to +1.06 mm), remaining within clinically acceptable bounds. Detailed results are presented in Tables 3 and 4.

**TABLE 3:** Agreement segmentation morphological parameters—Internal test set (*n* = 42).

Morphological parameter	AI (mm)	Manual (mm)	Mean difference	95% limits of agreement	ICC (95% CI)
Maximum diameter	7.85 ± 3.52	7.76 ± 3.41	+0.09	[−0.68, +0.86]	0.941 [0.918, 0.958]
Neck width	4.21 ± 1.88	4.08 ± 1.79	+0.13	[−0.72, +0.98]	0.918 [0.889, 0.940]
Neck‐to‐dome ratio	0.556 ± 0.13	0.542 ± 0.11	+0.014	[−0.098, +0.126]	0.902 [0.869, 0.928]
Aspect ratio	1.308 ± 0.38	1.272 ± 0.34	+0.036	[−0.198, +0.270]	0.886 [0.848, 0.915]

*Note:* Agreement assessed using Bland–Altman analysis and intraclass correlation coefficient (ICC, two‐way mixed effects model, absolute agreement).

**TABLE 4:** Agreement segmentation morphological parameters—Independent external validation cohort (*n* = 21).

Morphological parameter	AI (mm)	Manual (mm)	Mean difference	95% limits of agreement	ICC (95% CI)
Maximum diameter	8.31 ± 4.18	8.19 ± 4.05	+0.12	[−0.82, +1.06]	0.928 [0.895, 0.951]
Neck width	4.35 ± 2.12	4.20 ± 2.01	+0.15	[−0.89, +1.19]	0.905 [0.868, 0.932]
Neck‐to‐dome ratio	0.561 ± 0.15	0.544 ± 0.13	+0.017	[−0.118, +0.152]	0.889 [0.848, 0.920]
Aspect ratio	1.321 ± 0.42	1.280 ± 0.38	+0.041	[−0.228, +0.310]	0.872 [0.827, 0.907]

*Note:* Agreement assessed using Bland–Altman analysis and intraclass correlation coefficient (ICC, two‐way mixed effects model, absolute agreement).

### 3.2 Detailed Analysis of Model Performance

Analysis by Morphological Complexity Index (MCI) showed a Dice coefficient of 0.891 ± 0.02 and PED selection accuracy of 94.9% for regular morphology (MCI < 0.3, 34.3% of cases), 0.873 ± 0.03 and 92.1% for moderately complex morphology (0.3 ≤ MCI < 0.6, 47.1%), and 0.848 ± 0.04 and 87.2% for highly complex morphology (MCI ≥ 0.6, 18.6%). By neck‐width ratio (neck width/maximum aneurysm diameter), narrow‐neck aneurysms (< 0.5, 53.5%) had a Dice coefficient of 0.883 ± 0.02 and PED selection accuracy of 93.4%, while wide‐neck aneurysms (≥ 0.5, 46.5%) recorded 0.865 ± 0.03 and 89.7%. Parent vessel curvature analysis revealed proximal landing‐zone offsets of 1.32 ± 0.41 mm for straight segments (< 0.1 mm^−1^, 35.8%), 1.55 ± 0.48 mm for curved segments (0.1–0.2 mm^−1^, 42.9%), and 1.85 ± 0.57 mm for highly curved segments (> 0.2 mm^−1^, 21.3%).

Stratified analysis by aneurysm multiplicity revealed statistically significant performance differences between single and multiple aneurysm cases (Table 5). Single aneurysm cases achieved a segmentation Dice coefficient of 0.882 ± 0.03 and PED classification accuracy of 93.2% ± 2.1%, compared with 0.856 ± 0.04 and 88.5% ± 3.8% for multiple aneurysm cases (*p* = 0.041 and *p* = 0.023, respectively). Modest but significant increases were also observed in diameter prediction error (0.22 ± 0.09 vs. 0.29 ± 0.13 mm, *p* = 0.031), length prediction error (1.28 ± 0.42 vs. 1.58 ± 0.55 mm, *p* = 0.044), and landing zone offsets in multiple aneurysm cases.

**TABLE 5:** Stratified performance analysis between single aneurysms and multiple aneurysms—Internal test set (*n* = 42).

Metric	Single aneurysm	Multiple aneurysms	*p*
Segmentation dice coefficient	0.882 ± 0.03	0.856 ± 0.04	0.041*
HD95 (mm)	4.2 ± 1.5	5.1 ± 1.8	0.038*
PED size classification accuracy (%)	93.2 ± 2.1	88.5 ± 3.8	0.023*
Diameter prediction error (mm)	0.22 ± 0.09	0.29 ± 0.13	0.031*
Length prediction error (mm)	1.28 ± 0.42	1.58 ± 0.55	0.044*
Proximal landing zone offset (mm)	1.45 ± 0.44	1.72 ± 0.52	0.048*
Distal landing zone offset (mm)	2.28 ± 0.65	2.68 ± 0.78	0.042*

*Note:* *p*‐values based on Mann–Whitney *U* test.

Abbreviations: HD95, 95th percentile Hausdorff distance; PED, pipeline embolization device.

* *p* < 0.05.

### 3.3 Multimodal Fusion and Feature Contribution Analysis

Using image features alone yielded an accuracy of 79.4% (contribution 34.2%, feature importance score 0.82). Geometric features alone achieved 75.6% accuracy (contribution 28.5%, importance 0.78). Knowledge, tabular, and temporal features contributed 15.6%, 10.3%, and 11.4%, respectively. Ablation studies showed accuracy drops of 12.4% upon removing image features, 8.2% for geometric features, 4.3% for knowledge features, 2.5% for tabular data, and 3.0% for temporal data. Tensor decomposition‐based multimodal fusion outperformed traditional methods: simple concatenation (86.3%), weighted averaging (87.8%), attention‐weighted fusion (88.5%), bilinear pooling (89.2%), and tensor decomposition (91.8%).

Visualization of modality interaction tensors revealed complex interaction patterns, with high‐frequency interactions concentrated in image‐geometric (32.5%), geometric‐knowledge (24.8%), and geometric‐temporal (18.7%) modality pairs. Interaction strength correlated positively with case complexity (*r* = 0.68, *p* < 0.001), indicating greater reliance on multimodal fusion in complex cases.

The visualization of feature clustering showed scattered, indistinct class boundaries in the pre‐fusion modality feature spaces. After fusion, clustering improved significantly, with a 42.3% reduction in intra‐class distance and a 35.7% increase in inter‐class distance, validating the efficacy of tensor decomposition fusion. In high‐dimensional feature space, 12 distinct PED size clusters emerged with clear boundaries, further confirming the success of the fusion process.

### 3.4 Statistical Analysis and Evaluation of Clinical Applications

On the independent set of 21 clinical cases, the NeurAneuNet system's primary PED recommendation demonstrated an accuracy of 95.2% (20/21) when compared to the optimal standard (Table 1). The cohort includes: 2 cases of microaneurysms (< 3 mm) including one patient with two adjacent microaneurysms, 10 cases of small aneurysms (3–5 mm), 6 cases of medium‐sized aneurysms (5–10 mm), 2 cases of large aneurysms (10–25 mm), and 1 case of giant aneurysm (> 25 mm).

A total of 6 physicians and 21 cases were included in the planning experiment, yielding 126 paired assessments (6 physicians × 21 cases) under the No‐AI and AI conditions. Overall, AI assistance significantly reduced planning time (Table 2). When pooling all physicians, the mean planning time decreased from 672 ± 225 s in the No‐AI condition to 371 ± 51 s in the AI‐assisted condition (*p* < 0.01). The reduction was most pronounced for junior physicians, whose planning time decreased from 933 ± 76 to 381 ± 51 s.

The subjective workload assessed by NASA‐TLX was also lower with AI assistance (Table 2). The mean NASA‐TLX score dropped from 33 ± 8 to 21 ± 5 for all physicians (*p* < 0.01). This reduction was consistent across all seniority levels, with junior physicians reporting the largest absolute decrease (from 41 ± 6 to 24 ± 4).

The agreement between the physician's selected PED and the optimal standard improved with AI assistance (Table 2). The overall agreement rate increased from 83.3% (105/126 selections) to 96.0% (121/126 selections) (*p* < 0.01). This improvement was particularly notable for intermediate and junior physicians. The agreement rate for intermediate physicians increased from 80.9% (34/42) to 95.2% (40/42), and for junior physicians from 69.0% (29/42) to 92.9% (39/42). All selections made by senior physicians were correct under both conditions.

## 4 Discussion

This study introduces NeurAneuNet, a system integrating multimodal deep learning and knowledge enhancement to achieve end‐to‐end prediction of PED size selection and landing‐zone placement from 3DRA images. We demonstrated its superior performance across multiple tasks, including aneurysm segmentation, geometric feature extraction, PED size selection, and landing‐zone guidance.

In terms of overall segmentation accuracy, NeurAneuNet's Dice coefficient (0.874 ± 0.03) was comparable to TransBTS (0.878 ± 0.02) but significantly outperformed other methods [21, 22], including nnFormer (0.863 ± 0.03), Swin‐UNETR (0.869 ± 0.03), ResUNet++ (0.845 ± 0.03), and U‐Net3D (0.826 ± 0.04). For boundary precision, NeurAneuNet's 95th percentile Hausdorff distance (HD95; 4.5 ± 1.6 mm) was 8.2% lower than that of TransBTS (4.9 ± 1.6 mm) [23, 24]. The system achieved clinical threshold compliance rates of 97.3% for overall Dice ≥ 0.85, 94.2% for PED classification accuracy ≥ 93%, 97.5% for diameter error ≤ 0.25 mm, 93.2% for proximal landing‐zone offset ≤ 1.5 mm, and 89.7% for distal landing‐zone offset ≤ 2.5 mm. These metrics surpassed existing methods, meeting clinical utility standards.

We observed higher prediction accuracy for short‐length PEDs compared to long‐length PEDs, likely due to the more complex vascular anatomy navigated by longer PEDs, which may span variable geometries or branch points. NeurAneuNet's millimeter‐scale diameter prediction precision (error 0.24 ± 0.10 mm) is critical for precise PED selection. A 0.5 mm error could lead to inappropriate model selection, thereby increasing treatment risks. This precision was enabled by dual‐path attention mechanisms and knowledge‐enhanced tensor decomposition. Analysis of prediction patterns revealed three key trends: (1) proximal landing‐zone predictions were consistently more accurate than distal ones; (2) larger PED diameters were associated with increased positioning errors; and (3) longer PEDs exhibited greater positioning errors. These findings align with clinical experience, as large‐diameter and long PEDs are typically used in complex anatomies, posing greater positioning challenges. For vessels with diameters < 3.5 mm, the model's proximal offset (1.25 ± 0.39 mm) neared expert‐level performance, offering significant value for delicate vascular interventions. The reduced performance observed in cases with multiple aneurysms may be attributable to competing segmentation signals from concurrent lesions, which can increase ambiguity in parent vessel centerline extraction and subsequently propagate errors through the geometric feature extraction and multimodal fusion pipeline. Future studies should investigate dedicated attention modules or lesion‐specific segmentation strategies to address this limitation. In addition, morphological parameter analysis comparing AI‐derived and manual segmentations yielded ICC values of 0.886–0.941 on the internal test set and 0.872–0.928 on the external validation cohort, indicating that NeurAneuNet generates anatomically consistent measurements that support reliable PED treatment planning across diverse aneurysm morphologies. Notably, distal landing‐zone prediction accuracy appeared more sensitive to PED length, possibly because longer PEDs traverse greater vascular curvature and larger diameter differences between proximal and distal vessel segments.

Analysis of prediction failures (Figure 4b) identified three primary categories: complex morphologies (62.3%, Figure S1a), indistinct boundaries (24.5%, Figure S1b), and microaneurysms (13.2%, Figure S1c). These failure patterns may arise from different stages of the pipeline. Complex morphology and marked vessel tortuosity can destabilize centerline extraction and amplify downstream landing‐zone errors, whereas indistinct aneurysm boundaries may impair neck localization and parent‐vessel contour estimation. For microaneurysms, limited voxel‐level representation and partial‐volume effects can make small absolute segmentation errors clinically relevant, particularly when the estimated vessel diameter lies near a PED size transition threshold. Representative cases and stage‐specific error propagation pathways for these three failure categories are provided in the Supporting Information and Figure S1.

Cases with diameters near adjacent model thresholds (±0.1 mm) showed a 3.5% reduction in accuracy, highlighting boundary cases as a system challenge. In cases with concurrent high surface‐area ratio (> 1.2), high curvature variance (standard deviation > 0.15), and low sphericity (< 0.8), NeurAneuNet maintained an accuracy of 83.5%, demonstrating robustness for extreme cases. This adaptability stemmed from knowledge‐enhanced networks and adversarial training. Vascular bifurcations posed another challenge: aneurysms within 5 mm of a bifurcation (19.4% of cases) had reduced PED size accuracy (87.6%) and increased proximal offsets (1.78 ± 0.55 mm). Nevertheless, NeurAneuNet outperformed comparator methods at bifurcations (mean accuracy improvement of 5.8%), attributed to knowledge enhancement and multimodal feature analysis capturing bifurcation‐specific vascular characteristics.

Tensor decomposition‐based feature fusion significantly outperformed traditional methods, particularly for complex wide‐neck aneurysms, improving accuracy by 5.4%–9.8%. This underscores the importance of capturing high‐order feature interactions for complex morphology assessments [25]. Modality interaction analysis revealed dominant interactions in image‐geometric (32.5%), geometric‐knowledge (24.8%), and geometric‐temporal (18.7%) pairs. Interaction strength correlated with case complexity (*r* = 0.68, *p* < 0.001), confirming greater reliance on multimodal fusion in complex cases. For micro and small aneurysms (< 5 mm), image (42.3%) and geometric (31.5%) features predominated, whereas knowledge features contributed 21.4% for large, complex aneurysms, aligning with clinical practice where expert experience is critical for complex cases. Synergistic effects were pronounced when combining image and geometric features [26], boosting accuracy from 79.4% and 75.6% individually to 86.5%, exceeding additive effects. This nonlinear complementarity was quantified by synergy indices > 1 across multiple modality pairs.

Despite its strong performance, NeurAneuNet has limitations requiring future attention. First, although NeurAneuNet demonstrated robust performance in the current datasets, its generalizability remains to be further established. The training, validation, and clinical evaluation data were derived from a single institutional healthcare system, with limited diversity in geography, patient population, imaging protocols, scanner platforms, and interventional practice patterns. Moreover, despite the inclusion of giant and microaneurysm subtypes in the expanded external validation cohort, the number of such morphologically challenging cases remained limited. Therefore, further multicenter, prospective validation across heterogeneous clinical settings and enriched challenging aneurysm subgroups is warranted to confirm the broader applicability of NeurAneuNet. Second, errors increased for rare anatomical scenarios, such as giant aneurysms and vessels with significant diameter variations, due to limited sample sizes. Third, the system currently leverages only geometric and imaging features and excludes hemodynamic simulations, which limits its ability to predict post‐treatment intra‐aneurysmal thrombosis and endothelial remodeling. Fourth, this study did not directly evaluate post‐procedural safety outcomes, including device malapposition, vessel dissection, in‐stent thrombosis, thromboembolic events, or long‐term aneurysm occlusion. Therefore, the potential impact of NeurAneuNet on procedural safety and patient outcomes requires further validation. Finally, the PEDs in this study are all from Medtronic's Pipeline Flex or Pipeline Shield. Due to differences in mechanical properties across FD brands, retraining is necessary for application to other devices.

To address these limitations, future research should pursue six directions: (1) collect multicenter, heterogeneous imaging and clinical data from diverse populations, incorporating domain adaptation and federated learning to enhance cross‐center and cross‐device generalizability; (2) employ cross‐dataset transfer learning or WGAN‐based sample synthesis to augment data for rare anatomies, such as giant or multi‐sac aneurysms; (3) integrate computational fluid dynamics simulations or graph neural network‐driven hemodynamic prediction modules into the pipeline, creating a comprehensive image‐hemodynamic‐knowledge decision framework; (4) incorporate systematic postoperative imaging and longitudinal clinical follow‐up to assess device malapposition, dissection, thrombosis, aneurysm occlusion, and patient outcomes; (5) conduct pilot deployments in real‐world clinical settings, gathering interventionalist feedback and patient outcome data to iteratively refine the model; (6) incorporate multi‐brand FD datasets to improve device generalizability.

## 5 Conclusions

This study presents NeurAneuNet, an end‐to‐end intelligent decision support system that integrates multimodal deep learning with knowledge enhancement. By combining 3DRA image segmentation, geometric feature extraction, knowledge graph embedding, tensor decomposition‐based multimodal fusion, and high‐order KAN, NeurAneuNet achieves fully automated guidance for PED size selection and landing‐zone placement from 3DRA images. The system demonstrates clinically relevant accuracy, efficiency, and applicability, providing robust intelligent support for intracranial aneurysm interventions. Furthermore, it offers a generalizable framework for artificial intelligence‐driven solutions to other complex medical decision‐making challenges.

## Author Contributions

Zhihong Wen, Shengli Guo, and Yulin Peng: Contributed equally to this work. Zhihong Wen and Yulin Peng contributed to investigation and methodology. Shengli Guo contributed to writing – original draft, visualization, and handled manuscript submission. Luokai Huangfu, Hao Zhao, Hao Gao, and Taoyi Ni gathered and organized the clinical case data. Jianning Zhang supervised the project. Jiayu Liu contributed to data visualization and writing – review and editing. Xiangpeng Liu and Yongping Liang contributed to study conceptualization and project management.

## Funding

The authors have nothing to report.

## Ethics Statement

The authors confirm that any aspect of the work covered in this manuscript that has involved patients with disorders of aneurysm has been conducted with the ethical approval approved by the Ethics Committee of PLA General Hospital (S2025‐751‐01). This study follows the guidelines of the Declaration of Helsinki for humans. Informed consent was obtained from all individual participants included in the study.

## Conflicts of Interest

The authors declare no conflicts of interest.

## Supporting information


**Figure S1:** Three primary categories of prediction failures: Complex morphology—severe vessel tortuosity leading to proximal landing zone underestimation and insufficient PED length selection (a); Indistinct boundaries—morphologically complex aneurysm with partial vessel wall adherence leading to neck misidentification and proximal landing zone displacement (b); Micro‐aneurysm—failure to detect a concurrent small aneurysm leading to overestimated parent vessel diameter and erroneous PED diameter selection(c).

**Data S1:** cns71047‐sup‐0001‐Supinfo01.docx.

## Data Availability

The data supporting the findings of this study are not publicly available due to patient privacy and institutional ethical restrictions. De‐identified data may be made available from the corresponding authors upon reasonable request for academic and collaborative purposes, subject to approval by the Ethics Committee of the Chinese PLA General Hospital and completion of an appropriate data use agreement.
