# Supplementary figures and images for: Multimodal Deep Learning and Knowledge‐Enhanced Intelligent Decision Support System for Pipeline Embolization Device Size Selection in Intracranial Aneurysm Treatment
Source: CNS Neurosci Ther. 2026 Jul 22;32(7):e71047. doi: 10.1002/cns.71047 (PMC13390615; doi:10.1002/cns.71047)

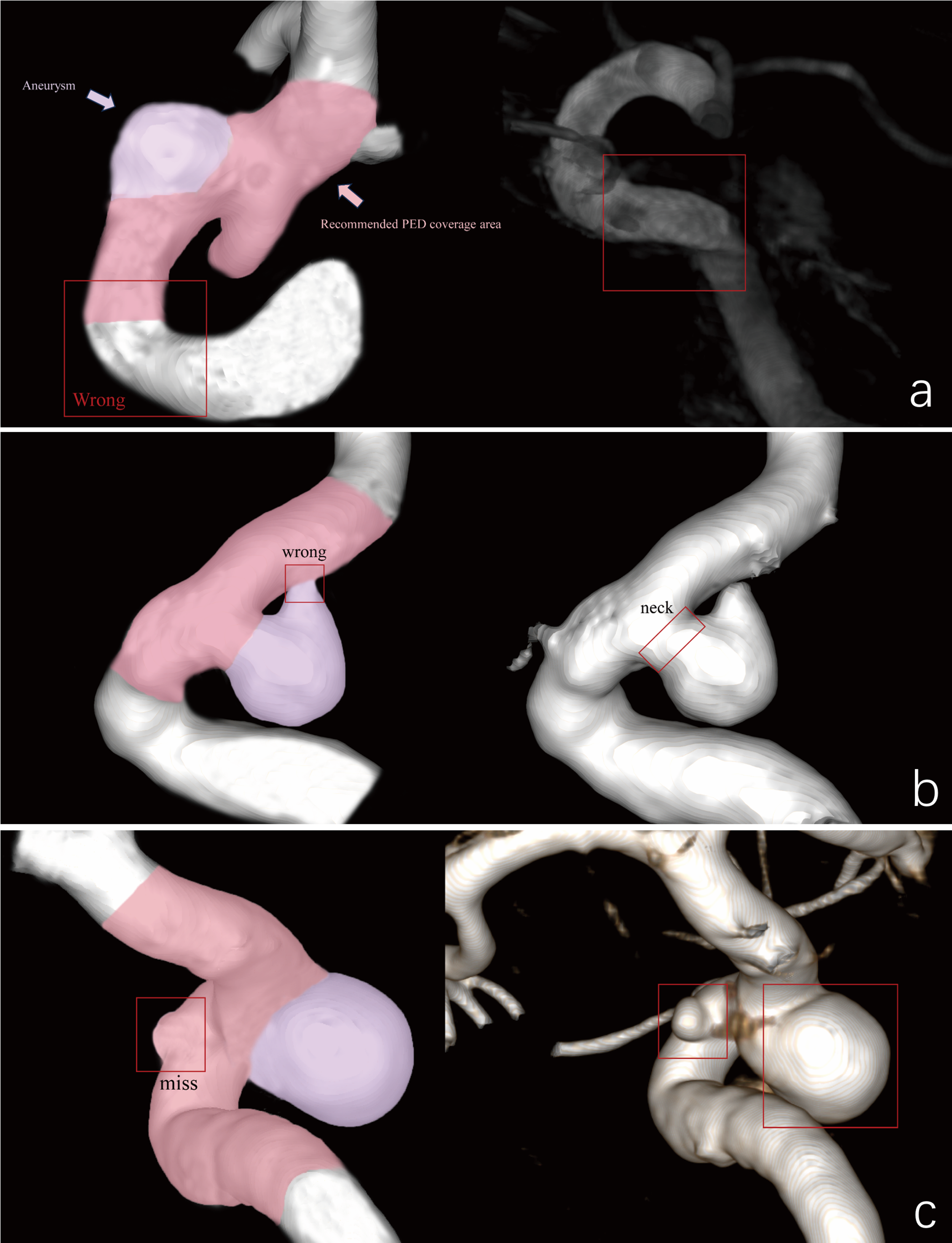

Supplement: Supplementary file 1 — Figure S1: Three primary categories of prediction failures: Complex morphology—severe vessel tortuosity leading to proximal landing zone underestimation and insufficient PED length selection (a); Indistinct boundaries—morphologically complex aneurysm with partial vessel wall adherence leading to neck misidentification and proximal landing zone displacement (b); Micro‐aneurysm—failure to detect a concurrent small aneurysm leading to overestimated parent vessel diameter and erroneous PED diameter selection(c). [file CNS-32-e71047-s002.zip › Supplementary Figure.1.tif]
