# Supplementary material for: Multimodal Deep Learning and Knowledge‐Enhanced Intelligent Decision Support System for Pipeline Embolization Device Size Selection in Intracranial Aneurysm Treatment
Source: CNS Neurosci Ther. 2026 Jul 22;32(7):e71047. doi: 10.1002/cns.71047 (PMC13390615; doi:10.1002/cns.71047)
Supplement: Supplementary file 2 — Data S1: cns71047‐sup‐0001‐Supinfo01.docx. [file CNS-32-e71047-s001.docx]

**Multimodal Deep Learning and Knowledge-Enhanced Intelligent Decision Support System for Pipeline Embolization Device Size Selection in Intracranial Aneurysm Treatment**

Zhihong Wen^1#^, Shengli Guo^2#^, Yulin Peng^1#^, Yakun Chen^2^, Luokai Huangfu^2^, Hao Zhao^3^, Hao Gao^2^, Taoyi Ni^2^, Jianning Zhang^2^, Xiangpeng Liu^1*^, Jiayu Liu^2*^, Yongping Liang^2*^

1 College of Information, Mechanical & Electrical Engineering, Shanghai Normal University, 100 Haisi Road, Shanghai, 201418, China

2 Department of Neurosurgery, the First Medical Center, Chinese PLA General Hospital, Fuxing Road 28, Beijing 100853, China

3 Department of Neurosurgery, the Six Medical Center, Chinese PLA General Hospital, Fucheng Road 6, Beijing 100048, China

#Zhihong Wen, Shengli Guo and Yulin Peng contributed equally to this work.

*Corresponding authors: Xiangpeng Liu, Jiayu Liu and Yongping Liang

E-mails: [xliu@shnu.edu.cn](mailto:xliu@shnu.edu.cn); [liujiayu@pku.edu.cn](mailto:liujiayu@pku.edu.cn); [liangyp199601@163.com](mailto:liangyp199601@163.com)

Case 1: Complex morphology — severe vessel tortuosity leading to proximal landing zone underestimation and insufficient PED length selection. In this case, pronounced vessel tortuosity represented a complex morphological configuration that destabilized centerline extraction at the geometric feature extraction stage, producing systematic underestimation of the effective curvilinear path length between the proximal landing zone and the aneurysm neck. As a result, the proximal landing zone was predicted too close to the aneurysm neck, and the system recommended a PED of insufficient length to achieve adequate proximal coverage. The underlying technical mechanism involves curvature-induced path length compression in the Marching Cubes-derived centerline representation: under high vessel curvature, the centerline algorithm underestimates the true curvilinear distance, directly causing the system to select a shorter device than clinically required. Segmentation quality remained within acceptable bounds in this case, confirming that the error was confined to the geometric feature extraction and downstream decision stages rather than the segmentation module.

Case 2: Indistinct boundaries — morphologically complex aneurysm with partial vessel wall adherence leading to neck misidentification and proximal landing zone displacement. In this case, the aneurysm exhibited complex dome morphology with a portion of the aneurysm wall partially adherent to the distal parent vessel, creating an anatomically ambiguous neck boundary that is characteristic of the indistinct boundary failure category. This adherence caused the segmentation module to misidentify the true aneurysm neck position, displacing it distally toward the adherent region, as the intensity and geometric features of the adherent wall segment closely mimicked those of the parent vessel lumen, preventing the dual-path attention U-Net++ from disambiguating the true neck boundary. Consequently, the proximal landing zone prediction was anchored to the erroneously identified neck rather than the true anatomical neck, resulting in a proximal landing zone that was too short and insufficiently proximal to the actual aneurysm origin. This segmentation-stage boundary misidentification propagated directly into the geometric feature extraction module, where the incorrectly estimated neck position served as the reference anchor for proximal landing zone calculation, amplifying the initial error into a clinically significant placement deviation.

Case 3: Micro-aneurysm — failure to detect a concurrent small aneurysm leading to overestimated parent vessel diameter and erroneous PED diameter selection. In this case, the patient presented with two concurrent aneurysms: one medium aneurysm and one micro-aneurysm. The system successfully identified and segmented the medium aneurysm; however, the micro-aneurysm was not recognized as a distinct lesion at the segmentation stage, as its dimensions approached the voxel resolution limit (0.5×0.5×0.8 mm) and its appearance was misclassified as a focal vessel wall irregularity rather than a discrete aneurysmal outpouching. This misclassification caused the undetected micro-aneurysm to be incorporated into the parent vessel segmentation mask, increasing the local vessel diameter measurement at the geometric feature extraction stage. The erroneously elevated parent vessel diameter estimate was subsequently propagated through the multimodal fusion module into the KAN prediction layer, where it biased the PED diameter recommendation toward a larger size category than was clinically appropriate, resulting in an incorrect PED diameter selection. This case illustrates how a resolution-limited segmentation failure for a secondary lesion can indirectly corrupt the primary morphometric measurements used for device sizing.

**Figure Legends**

**Supplementary Figure 1.** Three primary categories of prediction failures: Complex morphology — severe vessel tortuosity leading to proximal landing zone underestimation and insufficient PED length selection (a); Indistinct boundaries — morphologically complex aneurysm with partial vessel wall adherence leading to neck misidentification and proximal landing zone displacement (b); Micro-aneurysm — failure to detect a concurrent small aneurysm leading to overestimated parent vessel diameter and erroneous PED diameter selection(c).
